# The Effect of Cultivation Conditions on Antifungal and Maize Seed Germination Activity of *Bacillus*-Based Biocontrol Agent

**DOI:** 10.3390/bioengineering9120797

**Published:** 2022-12-13

**Authors:** Vanja Vlajkov, Ivana Pajčin, Marta Loc, Dragana Budakov, Jelena Dodić, Mila Grahovac, Jovana Grahovac

**Affiliations:** 1 Faculty of Technology Novi Sad, University of Novi Sad, Bulevar cara Lazara 1, 21000 Novi Sad, Serbia; 2 Faculty of Agriculture, University of Novi Sad, Trg Dositeja Obradovića 8, 21000 Novi Sad, Serbia

**Keywords:** microbial biopesticide, plant growth promotion, agitation speed, aeration rate, laboratory bioreactor, bioprocess kinetics, *Bacillus*, *Aspergillus flavus*, aflatoxin

## Abstract

Aflatoxin contamination is a global risk and a concerning problem threatening food safety. The biotechnological answer lies in the production of biocontrol agents that are effective against aflatoxins producers. In addition to their biocontrol effect, microbial-based products are recognized as efficient biosolutions for plant nutrition and growth promotion. The present study addresses the characterization of the representative of *Phaseolus vulgaris* rhizosphere microbiome, *Bacillus* sp. BioSol021, regarding plant growth promotion traits, including the activity of protease, cellulase, xylanase, and pectinase with the enzymatic activity index values 1.06, 2.04, 2.41, and 3.51, respectively. The potential for the wider commercialization of this kind of product is determined by the possibility of developing a scalable bioprocess solution suitable for technology transfer to an industrial scale. Therefore, the study addresses one of the most challenging steps in bioprocess development, including the production scale-up from the Erlenmeyer flask to the laboratory bioreactor. The results indicated the influence of the key bioprocess parameters on the dual mechanism of action of biocontrol effects against the aflatoxigenic *Aspergillus flavus*, as well on maize seed germination activity, pointing out the positive impact of high aeration intensity and agitation rate, resulting in inhibition zone diameters of 60 mm, a root length 96 mm, and a shoot length 27 mm.

## 1. Introduction

Food safety issues threatening public health and economic stability worldwide are considered one of the leading global challenges. The international collaboration and export of food products influence the potential health risks not only inside but outside national borders [1]. Economic losses measured in USD billions caused by fungal pathogens confirm the magnitude of the existing problem. Representatives of the *Aspergillus* genus, which have the ability to produce secondary metabolites with intensive toxic effects, are characterized as a major threat among fungal pathogens [2]. The impact of climate changes additionally contributes to the unfavorable predictions in terms of the production of healthy crops, an important factor for the development and enhancement of the negative impacts of the aflatoxigenic *Aspergillus* species. Therefore, there is an inevitable need to intensify activities in the field of pathogen control strategy as a prerequisite for improving the quality and safety of food for animal and human use [3,4]. Different strategies have been employed so far to minimize the harmful effect of aflatoxins’ presence in agricultural production systems. Some of the most common, which showed limited success when applied independently, include agrochemicals usage, timely irrigation, and alternate cropping systems, while more promising results were obtained as a result of synergistic effects by combining the aforementioned techniques [5]. Speaking of biological solutions, researchers proposed the usage of nontoxigenic strains of *Aspergillus* as biocontrol agents, whose mechanism of action is based on the competition with toxigenic ones in terms of infection sites and nutrients [4]. However, among different conventional and innovative approaches, microbial biopesticides were recognized as one of the most promising and eco-friendly solutions to address the existing problem.

The representatives of the *Bacillus* genus have been recognized as a group of agriculturally important microorganisms defined as potent biocontrol agents and plant growth-promoting rhizobacteria (PGPR). Rhizobacteria act as growth promoters in a direct or indirect way through several mechanisms of action, including nitrogen fixation, nutrient solubilization and biosynthesis of phytohormones, antibiotics, hydrolytic enzymes, and siderophores, as well as through the induction of systemic plant resistance [6]. Members of the genus *Bacillus* are significant producers of biologically active molecules with inhibitory activity against phytopathogenic species and additional plant-beneficial properties, including the ability to support plant root colonization and act as immune stimulators [7]. Some of the biocontrol-related metabolic products include lipopeptides with antimicrobial and biosurfactant traits, antibiotics of other classes, and volatile organic compounds [8,9,10]. These extracellular metabolites could be separated or purified to be used as biocontrol agents themselves or could be produced during the interaction of the biocontrol strain with a target phytopathogen at the application site, pointing out the significance of microbial viability preservation during the complete production procedure, distribution, storage, and application of the microbial-based biocontrol products [11,12]. The production of phytohormones, such as indole acetic acid (IAA), is directly linked to nutrient availability, influencing soil quality and resulting in plant growth promotion [13,14]. The biosynthesis of hydrolytic enzymes is an important PGP characteristic contributing to the nutrient acquisition and competitive activity of the *Bacillus* strains against phytopathogens in terms of nutrient availability and growth space [15]. A number of studies have demonstrated that *Bacillus*-based products can improve several attributes of plant growth, including seed germination, shoot and root length, seedling biomass, and size of leaves [6,16].

Crop protection management is currently in the transitional phase, and the high potential of switching from the dominant usage of chemical pesticides to the more sustainable alternatives is recognized in the *Bacillus*-based biocontrol agents [11]. In addition to the beneficial effect regarding the aforementioned PGP activities, representatives of the *Bacillus* genus also have a biocontrol effect that contributes to their potential, and possible wider application in agricultural practice. Metabolic activity with an emphasis on the production of lipopeptides was defined as crucial factor in antimicrobial activity against different bacterial and fungal pathogens [8]. The most important cyclic lipopeptides produced by *Bacillus* species are members of the surfactin, iturin, and fengycin families. The primary mechanism of action, providing suppressive effects against a wide range of pathogens, includes interactions with their cell wall, causing significant changes in structure and permeability. Recent studies also confirmed the important role of lipopeptides in the colonization and persistence of *Bacillus* in the rhizosphere. [17]. In addition to their incontestable biocontrol potential, *Bacillus* species are characterized by their ability to replicate rapidly, resistance to adverse environmental conditions, and production of endospores, which makes them suitable candidates for the development of bioprocess solutions [15].

The overall potential regarding the positive impact on agricultural production confirms the importance of investigation and the development of commercially viable biological products for plant protection and nutrition based on PGP and biocontrol strains. However, detailed characterizations and understandings of the biocontrol and plant-beneficial activity routes of certain microorganisms are necessary in order to achieve maximal efficiency at the application site and realize the commercial potential of the final product [17]. In addition to defining the highly potent and fully characterized microbial strain in terms of PGP and biocontrol activity, it is of utmost importance to define sustainable and techno-economically feasible production processes for microbial biocontrol products to establish a basis for successful commercialization and market competitiveness. Speaking of the microbial biotechnology domain, increasing the production scale relies on the successive optimization sets performed at laboratory level, which define the critical factors necessary for a further scale-up to the manufacturing stage [18]. Performing bioprocesses at the level of a laboratory bioreactor is an important step in the development of bioprocess solutions for the production of microbial biocontrol agents and a starting point of the technology transfer to a larger-scale production. This bioprocess development step allows the simulation of production under conditions as close as possible to those on semi-industrial and industrial scales. A technology transfer from laboratory to higher production scales that will enable the commercialization of microbial products relies on first defining the optimal bioprocess parameters that favor the growth and metabolic activity of the selected producing microorganism used as a biocatalyst or, in this case, the active component of the microbial biocontrol product [19,20,21]. Generally speaking, agitation is a key parameter for efficient bioprocess operation, and its role is reflected in providing a homogeneous environment with a decisive influence on the degree of changes in hydrodynamic conditions, heat exchange, and mass transfer. On the other hand, aeration rate and dynamics define the level of oxygenation and additionally contribute to the homogenization of the cultivation medium [22,23,24]. A better understanding of the observed system’s behavior during microbial cultivation is possible through different modelling approaches, including studies aiming to perform bioprocess kinetics analysis that predominantly focus on microbial growth kinetics considering that the number of viable cells in the final microbial product is one of the main factors influencing its field efficiency [25,26,27]. Observing the bacterial growth and consumption of nutrients during cultivation is an important aspect of predictive microbiology, focusing on the key parameters defining the successful scaling of a certain bioprocess to the larger-scale production stages by simultaneously providing maximized bioprocess efficiency in terms of nutrients utilization [28]. All of the aforementioned aspects are important to be investigated at the lab scale in order to develop scalable, cost-efficient, and environmentally friendly bioprocess solutions for the production of highly efficient microbial biocontrol products.

According to the summarized literature data, the hypothesis of this study is that the representatives of the *Bacillus* genus are recognized as key biocatalysts from the perspective of the possibility for the development and commercial exploitation of bio-based products intended for usage in agriculture. The first aim of the study was to characterize the producing microorganism *Bacillus* sp. BioSol021 from the aspect of its plant growth promotion ability and additional beneficial effects on plant cultivation. As it was previously stated, the most challenging step in the technology transfer to the industrial production level is switching from external agitation and spontaneous aeration to internal agitation and controlled aeration conditions. Therefore, the second aim of the study was to investigate the impact of the key bioprocess parameters, including agitation rate and aeration intensity, on the production of *Bacillus*-based antimicrobial agents effective in the suppression of aflatoxigenic *A. flavus* performed in a 16 L stirred-tank bioreactor, as well as the effects of the final product the on maize seed germination activity. Besides the high-potential-producing microorganisms in terms of biocontrol and PGP activity, most studies are limited to investigations based on cultivations performed in small volumes, missing the data crucial for the production scale-up. Within this study, the research went one step forward, focusing not only on the potential of the selected biocatalysts as a beneficial strain but on the important factors of biotechnological production.

## 2. Materials and Methods

### 2.1. Producing Microorganism and Phytopathogens

The producing microorganism, *Bacillus* sp. BioSol021, was previously isolated from the rhizosphere of *Phaseolus vulgaris* and selected among 76 *Bacillus* strains as the most efficient biocontrol agent in *A. flavus* suppression [2]. The 16S rRNA sequencing and VITEK2 Compact System identification indicated the highest similarity of the producing microorganism to the members of the operational group *Bacillus amyloliquefaciens* (GenBank sequence accession number ON569805) [29]. The producing strain is deposited at the international depository service under the Budapest Treaty in the International Collection of Agricultural and Industrial Microorganisms, Faculty of Food Science, Szent István University, Budapest, Hungary, with the collection number 520/2021.

Phytopathogenic strains *A. flavus* SA2B and *A. flavus* PA2D were isolated from corn in the 2020 harvest season and identified to the species level based on morphological and biochemical identification, with proven ability to produce aflatoxins based on multiplex PCR (polymerase chain reaction) gene targeting and in vitro aflatoxins production screening in simulated favorable environmental conditions [2].

### 2.2. Screening of Plant Growth Promotion Traits

In order to better understand PGP potential of the producing strain *Bacillus* sp. BioSol021, several PGP parameters were screened, as described below. For the PGP screening procedure, *Bacillus* sp. BioSol021 was cultivated in Erlenmeyer flasks using the nutrient broth medium (Himedia Laboratories, Mumbai, India) for 48 h on a rotary shaker (170 rpm) at 28 °C, without or with addition of L-Trp (1.02 g/L) for the experiments aimed at indole compounds quantification. For the experiments requiring cell-free supernatant of the cultivation broth, centrifugation was performed to separate bacterial biomass (12,000 g, 10 min, 25 °C, Z 326 K, Hermle LaborTechnik GmbH, Wehingen, Germany). If not stated differently, the cultivation broth of *Bacillus* sp. BioSol021 was used in the PGP-assaying procedures.

#### 2.2.1. Protease Activity Screening

The proteolytic activity of the producing microorganism *Bacillus* sp. BioSol021 was assayed using the modified skim milk agar [30]. The medium composition is shown in Table 1. The pH value was adjusted to 7.0 ± 0.2 and the medium was sterilized by autoclaving (121 °C, 2.1 bar, 20 min). After inoculation of the skim milk agar plates using 1 µL of the *Bacillus* sp. BioSol021 cultivation broth prepared as described above, incubation was carried out for 120 h at 28 °C. The evaluation of the enzymatic activity was performed based on the appearance of the biomass growth and the clear zone around it. The extracellular enzymatic activity index (***EAI***) was calculated according to the following Equation (1) for protease and the following enzymes assayed:

(1) $$\mathrm{EAI}=\frac{\mathrm{diameter}\;\mathrm{of}\;\mathrm{biomass}\;\mathrm{growth}+\mathrm{diameter}\;\mathrm{of}\;\mathrm{halo}\;\mathrm{zones}}{\mathrm{diameter}\;\mathrm{of}\;\mathrm{biomass}\;\mathrm{growth}}$$

#### 2.2.2. Cellulase and Xylanase Activity Screening

The cellulase and xylanase activities of the producing microorganism *Bacillus* sp. BioSol021 were assayed using the media whose composition is shown in Table 2, containing carboxymethyl cellulose (CMC) or xylan as the main substrates [31]. Inoculation and incubation conditions were the same as described in Section 2.2.1. In order to provide better visualization of the clear zones around microbial growth indicating cellulase/pectinase activity, the media after the incubation were poured over using the Congo red solution (0.5% (*w*/*v*), 15 min) and afterwards the NaCl solution (1 M, 15 min). The measurement of the growth and halo zones was performed 10 min after removal of the NaCl solution.

#### 2.2.3. Pectinase Activity Screening

The pectinase activity of the producing microorganism *Bacillus* sp. BioSol021 was assayed using the medium whose composition is shown in Table 3 [32]. Inoculation and incubation conditions were the same as described in Section 2.2.1. Visualization of the halo zones was performed after pouring over the Gram’s iodine solution (5 min) and washing it with the distilled water.

#### 2.2.4. Indole Compounds Production

The colorimetric determination of indole compound content was performed by slightly modifying the method described by Syed-Ab-Rahman et al. [33]. Shortly, 1 mL of the *Bacillus* sp. BioSol021 cultivation broth supernatant was mixed with 2 mL of the Salkowski reagent (1.2% (*w*/*v*) FeCl_3_ in 7.9 M H_2_SO_4_) and incubated in a dark place at room temperature for 30 min. The appearance of pink color indicates the ability of the producing microorganism to produce indole compounds. After 30 min, the spectrophotometric measurement was performed at a wavelength of 535 nm (UV 1800, Shimadzu, Japan). Distilled water was used as a blank sample and the calibration curve was prepared using the indole acetic acid (IAA) standard (Sigma-Aldrich, Burlington, MA, USA).

### 2.3. Media Composition, Cultivation Conditions, and Bioprocess Monitoring

The optimal cultivation medium composition for *Bacillus* sp. BioSol021 cultivation was defined in our previous study [16] and it included (g/L): cellulose (5.0), (NH_4_)_2_SO_4_ (3.8), K_2_HPO_4_ (0.3), and MgSO_4_·7H_2_O (0.3). The inoculum preparation included transferring the loopful bacterial biomass to Erlenmayer flasks containing nutrient broth (50 mL) (HiMedia Laboratories, India) and cultivation on a rotary shaker at 28 °C, 170 rpm under spontaneous aeration for 24 h. The same experimental conditions were applied in the second step of inoculum preparation, when the liquid culture was transferred to the Erlenmayer flasks of higher volume (500 mL) containing the same commercial medium (150 mL) and cultivated for another 24 h. Inoculation of cultivation media for biosynthesis (10 L) was performed by adding 10% (*v*/*v*) of the prepared inoculum (1 L). The cultivation was carried out in a 16 L laboratory bioreactor (EDF–15.4_1, A/S Biotehniskais center, Latvia) in four experimental sets by varying agitation rate (between 100 and 300 rpm using three impeller Rushton turbines) and aeration rate (between 0.5 and 1.5 vvm—volume of sterile air/(volume of the medium·min)). The initial pH value of the medium was set to 7.0 ± 0.2 prior to sterilization by autoclaving, the bioprocess temperature was 28 °C, and the cultivation time 96 h. During the cultivation, the temperature was monitored using a temperature sensor (TP-206-CF-H1141-L450, Hans Turck GmbH & Co., Mülheim an der Ruhr, Germany), the oxygen saturation level using a pO_2_ electrode (OxyFerm FDA 425, Hamilton, Reno, NV, USA), and the pH value using a pH electrode (EasyFerm Bio HB K8 425, Hamilton, Reno, NV, USA). Detection of liquid and foam level in the bioreactor vessel was performed using conductometric sensors. Bioprocess control was performed using the BioRe SCADA software package (supervisory control and data acquisition, SCADA International, Silkeborg, Denmark). In addition to in-line instruments for bioprocess monitoring, off-line methods for cultivation broth analysis were employed by collecting samples at fixed defined time points. During the first day of cultivation (up to 24 h), sampling was performed every 3 h, while after that and until the end of the bioprocess, samples were collected every 6 h. A number of viable bacterial cells in *Bacillus* sp. BioSol021 cultivation broth samples was determined by the plate count method and given as CFU/mL (CFU—colony forming units). The carbon- and nitrogen-sources content were determined using standard analytical methods from Refs. [34,35], respectively.

### 2.4. Antimicrobial Activity Assay

Single-spore strains of test microorganisms (phytopathogenic strains *A. flavus* SA2B and *A. flavus* PA2D) are stored in sterile water and deposited at Microbial culture collection of Laboratory for detection of pathogens, pests, and weeds of Faculty of Agriculture, University of Novi Sad, Novi Sad, Republic of Serbia. *A. flavus* strains were center-point inoculated onto 5–2 agar medium and incubated 8–10 days at 31 °C. After the incubation time, the suspensions of test microorganisms were prepared by adding the fungal biomass in sterile saline to achieve the final spore concentration of 10^5^ CFU/mL (spores were harvested by gently scraping the agar surface with the sterile loop). Sabouraud maltose agar (SMA) media (Himedia Laboratories, Mumbai, India) were melted and tempered (50 ± 1 °C) and inoculated using 1 mL of the previously prepared fungal suspensions before pouring into the Petri dishes. The well diffusion method in triplicate tests was employed to evaluate the antimicrobial activity of the cultivation broth and cell-free supernatant samples (100 µL) collected during the cultivation of the producing microorganism, *Bacillus* sp. BioSol021, against the phytopathogenic strains. The cell-free supernatant samples were obtained by centrifugation, as explained in Section 2.2. The cultivation medium sample was used as a negative control. The incubation was performed at 30 °C for 96 h and was followed by the inhibition zone diameters measurement.

### 2.5. Seed Germination Activity Assay

Maize seeds (*Zea mays* subsp. *mays*) used for seed germination assay were surface sterilized using the chlorine bleach solution (6% (*v*/*v*), 1 min) and thoroughly washed using the sterile distilled water for 5 min. After drying, 25 seeds were placed in a Petri dish (90 mm) with the sterile filter paper at the bottom. Seeds were treated using 1 mL of the *Bacillus* sp. BioSol021 cultivation broth samples from the bioreactor cultivations or sterile distilled water (DW; negative control). Seeds incubation was performed in an incubator at 25 °C for 7 days, followed by enumeration of germinated seeds, as well as root and shoot length measurement.

### 2.6. Bioprocess Kinetics Analysis

Microbial growth kinetics were monitored during the cultivation of the production microorganism *Bacillus* sp. BioSol021 in a laboratory bioreactor. Microbial growth parameters (***N_0_*** (g/L)—initial biomass concentration of the producing microorganism, ***N_max_*** (g/L)—maximum biomass concentration of the production microorganism, and ***µ*** (1/h)—specific growth rate of the producing microorganism) were determined by applying Gompertz (2), logistic (3), and exponential plateau growth in Equation (4):

(2) $$N=N_{\max}\cdot {\left(\frac{N_{0}}{N_{\max}}\right)}^{e^{-\mu \cdot t}}\;$$

(3) $$N=\frac{N_{\max}\cdot N_{0}}{\left(N_{\max}-N_{0}\right)\cdot e^{-\mu \cdot t}+N_{0}}\;$$

(4) $$N=N_{\max}-\left(N_{\max}-N_{\max}\right)\cdot e^{-\mu \cdot t}$$

where ***N*** (g/L) represents the biomass concentration of the producing microorganism in time ***t*** (h).

### 2.7. Experimental Data Analysis

The experimental data were analyzed using ANOVA (analysis of variance) and Duncan’s multiple range test using Statistica 13.2 software (Dell, Round Rock, TX, USA). Distribution plots of the experiments, including maize seed germination, were generated in Python Jupyter Notebook 6.5.1 using matplotlib.pyplot and seaborn libraries. Bioprocess kinetic analysis was performed using GraphPad Prism software 8.4.3 (GraphPad Software, San Diego, CA, USA).

## 3. Results

### 3.1. PGP Traits of the Producing Strain Bacillus sp. BioSol021

**Figure 1:**
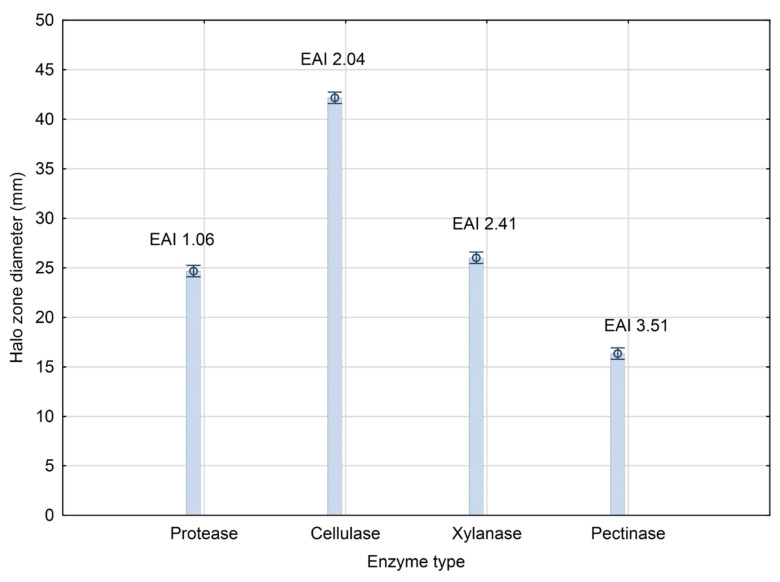
Screening of enzymatic activity of the producing strain *Bacillus* sp. BioSol021. ***EAI***—enzymatic activity index.

The potential of the producing strain *Bacillus* sp. BioSol021 for PGP was investigated in terms of enzymatic activity and ability to produce indole compounds. The results of enzymatic activity screening are presented in Figure 1, including the diameters of the overall halo zones and the growth diameter on the media containing the target enzyme substrates, together with the extracellular enzymatic activity index (***EAI***) showing the ratio of the enzymatic activity related directly to the extracellular enzyme production. The lowest value of the ***EAI*** was achieved in the case of medium for protease activity, indicating the lowest extracellular protease activity. The highest enzymatic activity was achieved in the case of cellulase screening medium, considering the joining growth and enzymatic hydrolysis diameter. However, the highest extracellular enzyme activity based on ***EAI*** value was achieved in the case of pectinase production.

The produced amount of indole compound in the nutrient broth-based cultivation medium by the producing strain *Bacillus* sp. BioSol021 was 6.76 ± 0.04 mg/L.

### 3.2. Microbial Growth Kinetics

Table 4 shows the growth parameters given by the three models representing the microbial growth curves of the producing microorganism *Bacillus* sp. BioSol021 cultivated in four experimental sets under different environmental conditions, including aeration intensity and agitation rate. The results indicated that predictive models fitted experimental data properly, with the high values of R^2^ above 0.97 in all cases. The best model fitness was achieved for the last experimental set by employing the logistic growth equation. On the other hand, the highest values for the specific microbial growth rate were obtained in the case of the first experimental set and, again, the highest value is 0.0554 (1/h), which was given by the logistic model. Speaking of the predicted values for the initial biomass concentration, the highest ***N_0_*** was obtained by the same kinetic model for the first experimental case. In contrast to other kinetics parameters, the exponential plateau model predicted the highest values for the maximum biomass concentration for the fourth experimental set.

**Figure 2:**
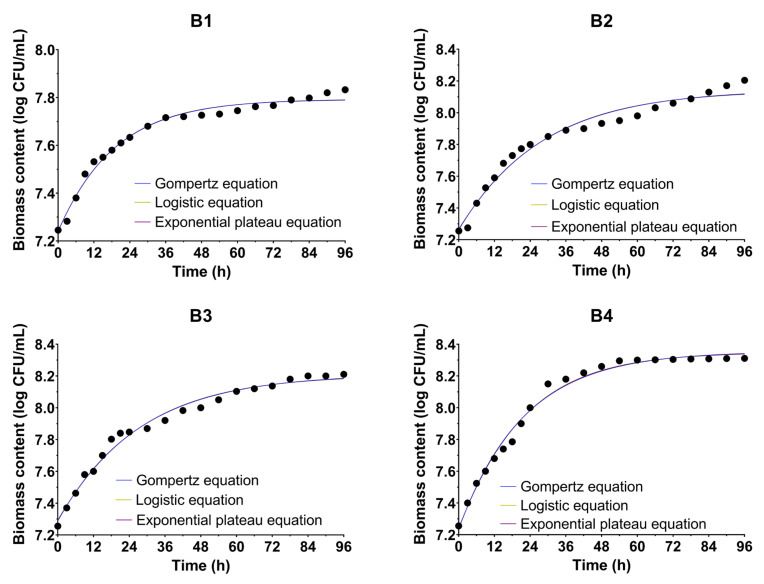
Growth curves of *Bacillus* sp. BioSol021 under different cultivation conditions fitted using the Gompertz, logistic, and exponential plateau equations. B1: 0.5 vvm, 100 rpm; B2: 0.5 vvm, 300 rpm; B3: 1.5 vvm, 100 rpm; B4: 1.5 vvm, 300 rpm.

Figure 2 presents the bacterial growth curves for all four experiments—cultivations of *Bacillus* sp. BioSol021 performed under different cultivation conditions—where the experimental data on cell numbers in certain time points during cultivation were fitted using the aforementioned Gompertz, logistic, and exponential plateau growth models. The results indicated a continuous increase in the producing microorganism’s cells concentration during the cultivation, with characteristic growth stages including lag, exponential, and stationary phases in each of the four investigated cases, as well as the satisfactory fitting of the experimental data using the previously explained kinetic models for microbial growth description.

### 3.3. Cultivation Course of Bacillus sp. BioSol021—Oxygen Saturation Level, pH Value, and Residual Nutrients (Cellulose, Total Nitrogen) Content

**Figure 3:**
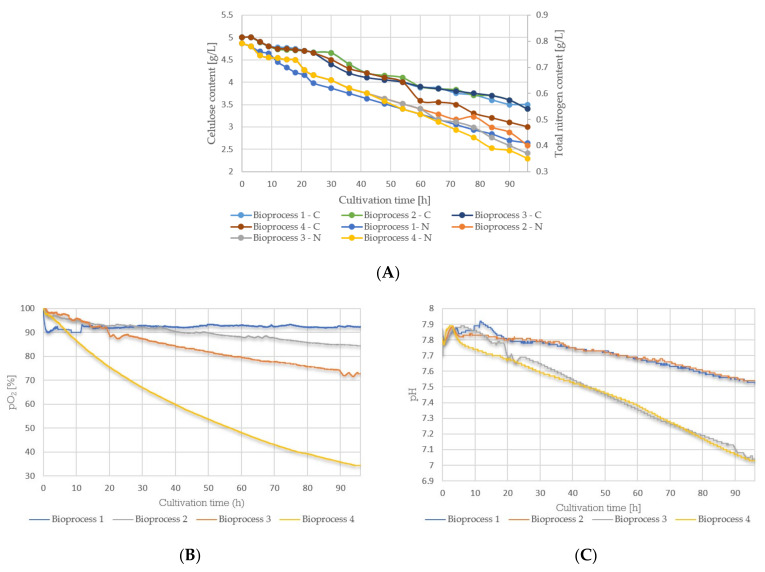
Cultivation of producing microorganism *Bacillus* sp. BioSol021 in 16 L laboratory bioreactor: (**A**) residual concentrations of nutrients (cellulose, total nitrogen); (**B**) oxygen saturation level; (**C**) pH value.

Figure 3A indicates the dynamics of the main nutrients’ consumption, including cellulose and total nitrogen. The results pointed out the evident decrease in residual concentrations of the main nutrients during the cultivation, with the highest consumption rate observed in the last experimental set. Figure 3B presents variations of the pH value during the cultivation, indicating a continual decrease in all four cases; however, it expressed the most in the last two experimental sets. Figure 3C shows the trend of oxygen saturation level changes, indicating the significant influence of a higher agitation rate and aeration intensity on the oxygen supply and, consequently, increased oxygen consumption.

### 3.4. Antimicrobial Activity of Bacillus sp. BioSol021 Cultivation Broth and Cell-Free Supernatant Samples against Aflatoxigenic A. flavus Isolates

**Figure 4:**
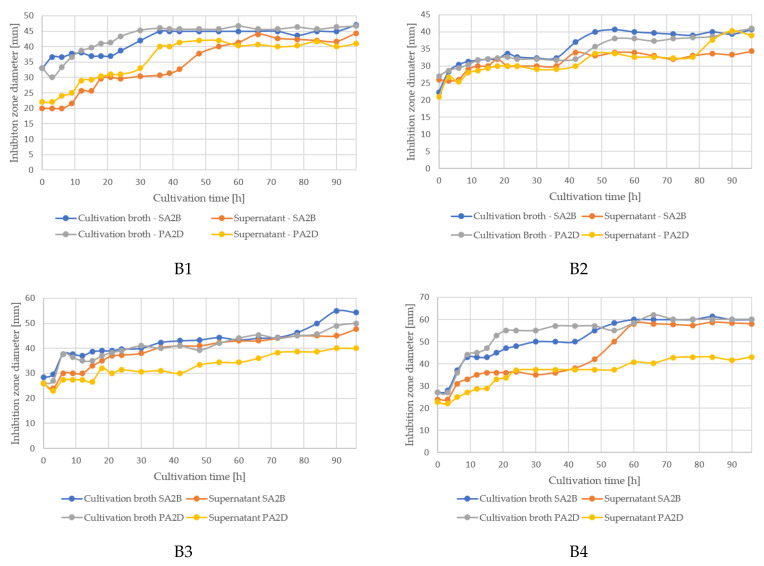
Antimicrobial activity assay of cultivation broth and cell-free supernatant samples of the producing microorganism *Bacillus* sp. BioSol021 against *A. flavus* strains SA2B and PA2D under different bioprocess parameters: B1: 0.5 vvm, 100 rpm; B2: 0.5 vvm, 300 rpm; B3: 1.5 vvm, 100 rpm; B4: 1.5 vvm, 300 rpm.

The monitoring of antagonistic activity against aflatoxigenic *A. flavus* SA2B and PA2D strains in experiments performed under different cultivation conditions is presented in Figure 4. The obtained results for cultivation broth and cell-free supernatant samples indicated a dualistic mechanism of action, implying the activity of producing microorganism cells present in cultivation broth samples, as well as the suppressive effect of metabolites as predominant active components in the cell-free supernatant samples. The highest antagonistic activity was achieved in the last experimental set, which is in line with the results presented in Figure 2, where the highest biomass concentration was obtained under the same cultivation conditions.

### 3.5. Maize Seed Germination Assay

**Figure 5:**
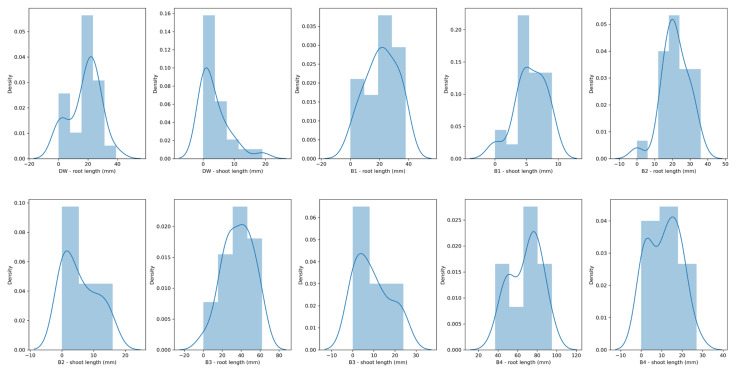
Distribution plots for root and shoot length of the germinated maize seeds treated with distilled water (DW) or *Bacillus* sp. BioSol021 cultivation broth samples produced under different cultivation conditions: B1: 0.5 vvm, 100 rpm; B2: 0.5 vvm, 300 rpm; B3: 1.5 vvm, 100 rpm; B4: 1.5 vvm, 300 rpm.

The efficiency of the *Bacillus* sp. BioSol021 cultivation broth in maize seed germination was assessed for cultivation broth samples obtained in four cultivations under different bioprocess conditions and compared with DW as the negative control. The distribution of the measured values of root and shoot length corresponds to normal distribution, as given in Figure 5.

The germination rates of maize seed treated with cultivation broth samples or DW, as well as maximal root and shoot length, are given in Table 5. A significant improvement in germination rate and maximal root and shoot length could be observed between negative control and cultivation broth-treated maize seeds, with an increasing trend following the increased agitation and aeration rate.

**Figure 6:**
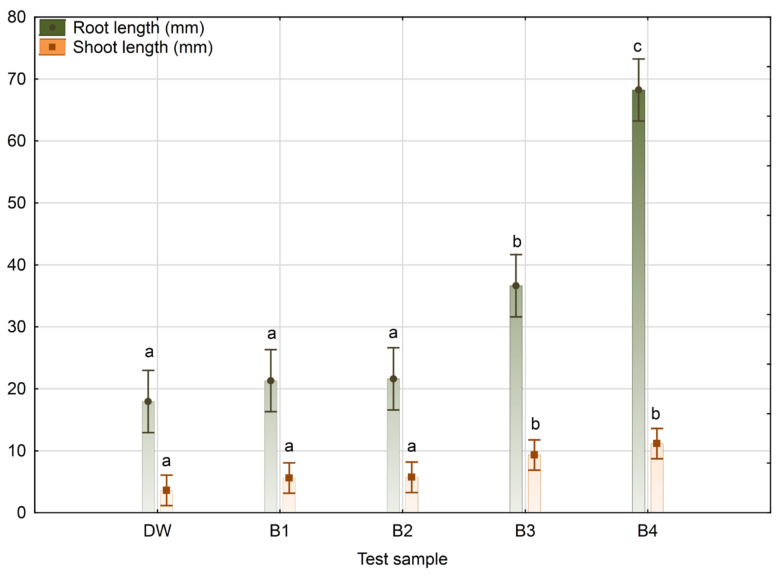
Mean values and standard deviations of maize root and shoot length for seeds treated with distilled water (DW) or *Bacillus* sp. BioSol021 cultivation broth samples produced under different cultivation conditions: B1: 0.5 vvm, 100 rpm; B2: 0.5 vvm, 300 rpm; B3: 1.5 vvm, 100 rpm; B4: 1.5 vvm, 300 rpm. Superscript letters designate different levels of statistical significance (with *p*-values over 0.05) according to Duncan’s multiple range test.

Mean values and standard deviations of root and shoot length, as well as homogenous groups of independent variables according to the Duncan’s multiple range test in terms of effect on maize seed root and shoot length, are given in Figure 6, and they follow the trend presented in Table 5. Namely, they point out significant differences between B3 and B4 grouped at the same level of significance compared with B1 and B2 cultivation broth samples closer to the negative control regarding the effect of maize root and shoot length.

## 4. Discussion

The bioprocess solution development for the production of a new microbial biopesticide involves a series of successive steps. In addition to the focus on the biological aspects of the potential biocontrol agent, the product development scheme includes an evaluation of the technical aspects of industrial-scale production, which is important from the point of view of potential commercialization. The central role in the development of bioprocesses is a selection of the most potent biocatalysts, which was performed in our previous research [2]. The selected producing microorganism *Bacillus* sp. BioSol021 has shown significant antagonistic potential in the suppression of several aflatoxigenic *Aspegillus flavus* strains. However, to develop a viable production strategy for biocontrol products planned to be used in the suppression of aflatoxigenic fungi, it is necessary to optimize medium composition, as well as to determine the optimal bioprocess conditions to maximize antagonistic activity against the target fungal pathogens. The following step in the bioprocess development has included the determination of optimal conditions in terms of qualitative and quantitative culture medium composition [36]. The next research step, included in this study, involves upgrading production to the laboratory level bioreactor and the optimization of key bioprocess parameters.

The determination of plant growth-promoting traits among *Bacillus* representatives was the focus of many research groups to define the capability of the investigated strains to be used as active components of beneficial products from the point of view of agricultural production [37,38,39,40]. In this context, the ability of plant-beneficial *Bacillus* strains to produce hydrolytic enzymes was previously reported by many research teams [41,42,43,44]. The importance of specific enzymes produced by *Bacillus* spp. lies in their involvement in nutrient and organic matter recycling, directly influencing the quality and state of the soil in terms of fertility [37]. The availability of nutrients has a beneficial effect on plant growth and nutrition but also a stimulative effect on soil microbiota. Microorganisms, as natural inhabitants of soil, are critical factors in the preservation of soil functionality through several functions, including soil formation, the decomposition of dead and decayed organic matter, cycling macro and micronutrients, and the removal/transformation of toxic chemicals into non-toxic forms [45]. An additional activity that is beneficial in the context of biocontrol is the mechanism of action that includes enzymatic activity involved in pathogens’ cell wall degradation [46]. Previous studies conducted research into the PGP and biocontrol traits of four *Bacillus* strains (*Bacillus simplex* 30N-5, *B. simplex* 11, *B. simplex* 237, and *B. subtilis* 30VD-1), including an evaluation of cellulase, xylanase, and pectinase production using agar assays [46]. In the present study, the production of extracellular hydrolytic enzymes, including protease, cellulase, xylanase, and pectinase, was determined in plate bioassays performed to not only investigate the ability of the producing microorganism *Bacillus* sp. BioSol021 to degrade complex substrates potentially present in the soil but also ways to enhance antifungal activity, with resulting extracellular ***EAI*** values of 1.06, 2.04, 2.41, and 3.51, respectively (Figure 1).

The investigated *Bacillus* sp. BioSol021 strain in this study showed proteolytic activity by joint biomass growth and a clear hydrolysis zone diameter of 24.17 ± 0.29 mm, which could be considered a moderate level of proteolytic activity. *Bacillus* strains exhibit a wide range of proteolytic activity, measured as a hydrolysis zone diameter, ranging from 1 to 2 mm to over 40 mm. Protease activity was evaluated in the study conducted by Pathak et al., in which clear zone diameters for 39 strains of the *Bacillus* genus were in range from 1 to 2.5 mm [47]. In the study published by Suberu et al., 48 *Bacillus* strains were isolated from soil samples, and 10 of them were defined as potential protease producers based on clear zones obtained on casein agar ranging from 33 to 49 mm [48]. The proteolytic bacteria were also isolated from leather waste, and the highest potential was observed for the representative of the *Bacillus* genus, with the extracellular ***EAI*** ranging from 2.27 to 2.45 and clear zones ranging from 34 to 44 mm depending on the pH value of the medium [49], suggesting the necessity to optimize the initial pH value of the cultivation medium or maintain the optimal pH value for protease production during the producing strain cultivation. The *Bacillus* species recognized as protease producers are *Bacillus cereus, Bacillus sterothermophilus, Bacillus mojavensis, Bacillus megaterium, Bacillus subtilis*, and *Bacillus amyloliquefaciens* [48,50]. The importance of the proteolytic activity, in addition to the decomposition of protein-based substrates present in the soil, is also associated with a cell wall-degrading capability, which is described as an additional biocontrol mechanism supporting the suppression of fungal pathogens [46]. The role of hydrolytic enzymes lies in the disruption of the structural integrity of the pathogens’ cell wall and cell lysis. Proteases are crucial factors in the lysis of the cell wall of fungal pathogens since their major constituents, including chitin and β-glucan fibrils, are embedded into the protein matrix. Additionally, some of the proteases are responsible for the inactivation of the extracellular enzymes of fungal pathogens [51]. The activity of proteases implies breaking down proteins into peptide chains and their constituent amino acids and consequently destroying the capacity of the pathogen’s functional enzymes to act on plant cells.

Cellulases hydrolyze the 1,4-β-D-glycosidic linkages in cellulose and have a significant role in nature by recycling this valuable polysaccharide. Taking into account the cellulose structure based on rigid crystalline microfibrils, the complete degradation requires a complex interaction between different cellulolytic enzymes, such as cellulose/endoglucanases, exo-cellobiohydrolase/exo-glucanases, and β-glucosidases, for synergistic action to convert cellulose into monomer units [51]. According to Gaur and Tiwari [52], hydrolysis zones larger than 10 mm in diameter suggest significant cellulolytic activity, consequently putting the *Bacillus* sp. BioSol021 strain in the group of highly efficient cellulase producers, even in the commercial medium nutrient broth, with a hydrolysis zone diameter of over 40 mm. The cellulase activity evaluated between 26 strains belonging to the *Bacillus* genus and the index of enzymatic activity up to six [53] suggested the necessity of medium and cultivation condition optimization in order to maximize the extracellular cellulolytic activity of the *Bacillus* sp. BioSol021 strain.

Pectinolytic activity is recognized as essential in the decay of dead plant material, especially when it comes to fruit and vegetable remains that are rich in pectin, resulting in the recycling of carbon compounds in the biosphere [54]. In a study by Algahtani et al. [55], pectinase production was evaluated by a clear zones measurement after the Petri plates were flooded with iodine solution, whereby 20 isolates were tested and 9 of them were positive for enzymatic activity, and the one with the highest potential was identified as *Bacillus subtilis*. The highest pectinase activity measured as a hydrolysis zone diameter ranging from 25 to 29 mm was achieved by the several *Bacillus* isolates from the rhizosphere of a medicinal plant, *Andrographis paniculata* [56]. *Bacillus* isolates from coffee pulp have shown an extracellular pectinase activity index ranging from 2.0 to 4.7 [57]. The pectinase production could be increased by a substrate-induced metabolism shift by including pectin-based substrates in the cultivation medium as one of the ways to maximize the production of pectinolytic enzymes.

Xylanases are one of the key enzymes responsible for the conversion of lignocellulose into fermentable sugars. Xylanase activity was previously reported for *Bacillus amyloliquefaciens* XR44A by selecting between 21 microorganisms screened on a solid medium [30]. Xylanolytic isolates isolated from soil with the highest enzymatic activity were identified as representatives of *Bacillus pumilus* and *Bacillus subtilis* species [58]. The clear zones around the bacterial growth were measured as indicators of hydrolysis activities in the study by Tamarez-Angelez et al., where 12 strains identified as members of the *Bacillus licheniformis* species and two *Bacillus subtilis* strains have shown enzymatic activity with clear zones of diameters up to 5 mm [59].

Indole acetic acid (IAA) is a common product of L-tryptophan metabolism, belonging to the group of phytohormones, and is defined as one of the most physiologically active auxins. The rhizospheric bacteria are recognized as dominant producers of more effective auxins compared with the isolates from the bulk soil. The ability of IAA synthesis is an additional criterion considered in plant-beneficial bacteria screening, taking into account its profound effect on plant growth. The plants’ treatment with products based on IAA-producing bacteria induces the proliferation of lateral roots and root hairs and an increased germination rate [60]. The phytostimulatory impact of IAA on plant growth improvement is explained by the stimulation of plant roots development and increasing the surface area to the volume ratio of the roots, which consequently results in a better uptake of water and nutrients, leading to higher yields [61]. Previous studies confirmed the ability of the representatives of the operational group *Bacillus amyloliquefaciens* to produce IAA, and investigations that included in vivo testing confirmed the contribution of the IAA-producing strains in plant growth promotion [38,62]. In the present study, the results indicated that the producing strain *Bacillus* sp. BioSol021 has the potential to produce an indole compound in a concentration of 6.76 mg/L, which makes it a suitable candidate for the further development of biotechnological products intended for usage in agriculture. Seven *Bacillus* isolates were tested for their ability to produce IAA, and the highest potential was observed for the strain *Bacillus* sp. BPR7 (17 mg/L) [63]. Another study conducted by Reetha et al., which evaluated the IAA-producing abilities of *Bacillus* isolates, has confirmed an IAA presence in a concentration of 12.67 mg/L [64].

Taking into account the obtained results in terms of PGP activity, in addition to its activity as a biocontrol agent, the investigated strain *Bacillus* sp. BioSol021 could also be considered as a promising active component of biofertilizer products. Similar PGP-related traits were found in several maize endophytic *Bacillus* isolates, which are the dominant members of the maize microbiome, especially in terms of cellulase, protease, pectinase, and indole compounds production [65]. Therefore, the PGP-beneficial traits confirmed for the *Bacillus* sp. BioSol021 strain could be successfully employed to strengthen the natural maize microbiome.

The monitoring of changes in biomass content during the *Bacillus* sp. BioSol021 cultivation was performed by determining the number of viable cells using plate counting as a standard microbiological technique. The trends shown in Figure 2 indicate typical growth curves characterized by the presence of three phases, which are expressed more or less depending on the certain experimental conditions applied. The first phase refers to the adaptation period of cells to the cultivation conditions—lag phase. A short lag phase or even its complete absence indicates a good adaptation of the producing microorganism cells to the applied conditions and the metabolizing of the present nutrients. It is obvious that in these particular cases, performing multiple passaging with inoculum preparation resulted in not only increasing the volume of the inoculum necessary for scaling the production to the bioreactor level but also in achieving its high quality. If a comparison was made between all experimental sets, it can be concluded that more intensive agitation is related to a slightly longer duration of the lag phase, which can be explained by higher cell stress due to more intensive mixing [66] as a consequence of the increased shear stress arising from internal mixing in the bioreactor compared with external mixing employed in the inoculum preparation stage. On the other hand, aeration is recognized as an important factor contributing to the better adaptation of the producing microorganism cells, due to better oxygen supply and intensified consumption in the first hours of cultivation [67]. After the lag phase, there is a period of intensive multiplication of the biomass, which can be noticed in all four experiments; however, it is expressed the most in the last experimental set. The positive but slightly slower growth trend continues during the stationary phase until the end of the bioprocess. The highest concentration of producing microorganism cells was reached under cultivation conditions characterized by the maximal values of the agitation rate (300 rpm) and aeration intensity (1.5 vvm).

The Gompertz, logistic, and exponential plateau equations were applied with the aim to fit the experimental data for biomass generation during the *Bacillus* sp. BioSol021 cultivation performed in four experimental sets under different cultivation conditions; however, they also determined the main kinetic parameters related to microbial growth, including the initial cell number ***N_0_***, maximal cell number ***N_max_***, and maximal specific growth rate ***µ***. The goodness of fit of the experimental data using the aforementioned equations was evaluated based on the determination coefficient R^2^. The kinetic model parameters and determination coefficient R^2^ for all three models are given in Table 4. The results indicated that the best model fitness, based on R^2^ value 0.9908, was achieved for the last experimental set, which had maximal values of aeration intensity and agitation rate, by employing the logistic growth equation. Comparing the R^2^ values between the selected models for each of the remaining experimental sets, it can be concluded that in all other cases, the highest values of R^2^ (confirming good model fitness) were observed for the exponential plateau equation, with the values for the first, second, and the third experiments being 0.9842, 0.9728, and 0.9867, respectively. When it comes to the kinetic parameters, the highest value of specific microbial growth rate is obtained in the case of the first experimental set, 0.0554 1/h, which is given by the logistic model, and is followed by the last experimental set, which had the value 0.050 1/h and was also given by the logistic equation. Speaking of the predicted values for the cell number, i.e., biomass concentration, the highest ***N_0_*** is obtained by the logistic model for the third experimental set, which is close to the experimental value of the initial biomass concentration of 7.25 CFU/mL; however, this is followed by a lower maximal biomass concentration ***N_max_*** compared with other models (8.204 CFU/mL), which is slightly different from the experimental value (8.21 CFU/mL) that is also better predicted by the exponential plateau model (8.212 CFU/mL). The logistic model predicted the highest values for the maximal biomass concentration for the fourth experimental set of 8.355 CFU/mL, which is close to the experimental value of 8.311 CFU/mL; however, slightly closer predictions were observed for the Gompertz (8.351 CFU/mL) and logistic models (8.347 CFU/mL).

Changes in the concentration of the main nutrients (carbon source and nitrogen source) during the bioprocess were monitored by determining the cellulose and total nitrogen content using standard analytical methods. Due to the consumption of nutrients in the biomass growth and metabolic activity of *Bacillus* sp. BioSol021, in all four experimental sets, there could be detected a continuous decrease in the residual concentrations of the aforementioned nutrients (Figure 3A). The initial stages of the cultivation are related to intensive cell growth and reproduction, while the further consumption of nutrients in the stationary growth phase is related to metabolic activity directed towards intensive intra- or extracellular metabolic product synthesis [68]. The results indicated that intensive agitation conditions led to a better distribution of nutrients in the total volume of the cultivation broth, making favorable conditions for their intensified consumption [69]. This statement is confirmed by the last experimental set results. Defined experimental conditions, including maximal aeration intensity and agitation rate, have enabled a better supply of nutrients to the producing microorganism cells, resulting in the most intensive consumption of carbon and nitrogen sources and leading to more intensive growth and reproduction, as well as the production of secondary metabolites [70]. On the other hand, the high residual concentrations of carbon and nitrogen sources indicated a necessity for the further improvement of the bioprocess parameters to ensure the better consumption of nutrients by using the same initial medium composition.

The changes in pH value during cultivation were monitored continuously, and the responses were detected every 60 s. Figure 3B shows the trend of changes in pH value in all four experimental sets. The continuous decrease was observed until the end of the bioprocess in all cases but it was more intensive in the last two experimental runs, which were characterized by maximal aeration intensity. The downward trend in pH value change is explained by the metabolic activity of the production microorganism, implying the production of acidic metabolites. In addition to the appropriate selection of components of the culture medium and the adequate level of oxygenation in the bioprocess, pH value is defined as an important parameter that determines the level of production of lipopeptides and other secondary metabolites, consequently defining the antagonistic potential of biocontrol agents [71]. The difference between the initial and final pH values during all four experimental sets did not exceed one pH unit. This pH range corresponds to ideal conditions for the growth and metabolic activity of the selected producing microorganism considering the previously reported optimal pH value ranges for *Bacillus* spp. [72,73].

Changes in dissolved oxygen content during cultivation were monitored continuously, and a response was detected every 60 s. Variations in examined bioprocess parameters, agitation rate, and aeration intensity were reflected most in the dissolved oxygen content. Figure 3C shows the trend of pO_2_ changes, indicating a continuous decrease in the amount of available oxygen during the entire duration of the bioprocess. The difference in the maximal value of the amount of available oxygen at the starting point of the bioprocess and the minimal value recorded at the end of cultivation increases from the first to the last experimental set. The results indicating oxygen consumption are in line with the previously described results for biomass growth [67]. A more drastic drop in the oxygen content is associated with greater needs of the producing microorganism due to entering the growth phase characterized by more intense metabolic activity. The experimental conditions in the last bioprocess favored the homogenization of the culture medium, mass transfer, and the supply of the cells with a sufficient amount of nutrients. Intensive aeration in combination with intensive agitation also provides an efficient dispersion of air bubbles and oxygen transfer to the bacterial cells [74]. In addition, intensive aeration enables the elimination of gaseous products generated during cultivation, providing more efficient bioprocesses and preventing inhibition due to the presence of gaseous metabolic by-products in the cultivation broth [66].

The determination of an antagonistic effect during the cultivation was monitored by testing the antimicrobial activity of the cultivation broth and cell-free supernatant samples against phytopathogenic and aflatoxigenic isolates, namely *A. flavus* SA2BSS and *A. flavus* PA2DSS. The mean values of the inhibition zone diameters (areas where there was no growth of phytopathogenic fungi) are shown in Figure 4. The obtained results indicated a double mechanism of action, which is characteristic for the representatives of the *Bacillus* genus [17]. The importance of the *Bacillus* representatives in the role of biocontrol agents is confirmed by a number of studies conducted by different research groups worldwide. The antifungal activity of *Bacillus velezensis* CE100 was determined in the study by Hong et al. [75], where its suppressive effect was evaluated against soil-borne fungal pathogens, including *Macrophomina phaseolina* isolate GL1310 and *Fusarium oxysporum* f. sp. *fragariae* isolate GL1080. The inhibitory effect was 64.7% and 55.2%, respectively. Another study included an investigation of the inhibitory effect of *Bacillus amyloliquefaciens* F9 against 16 phytopathogenic strains, including *A. flavus,* pointing out the antibiosis as a dominant biocontrol mechanism [76]. The biocontrol effect against the causer of celery powdery mildew disease *Erysiphe heracleid* was estimated using three *Bacillus* strains, namely *Bacillus subtilis, B. pumilus*, and *B. megaterium*. The inhibitory effect was evaluated on conidia germination and germ tube length by performing in vivo testing under field conditions, implying a high potential of biocontrol activity [77]. Another study included an evaluation of a biocontrol strategy based on *Bacillus amyloliquefaciens* HF-01, pointing out the high potential for the suppression of the postharvest decay of mandarin fruit caused by *Penicillium italicum, Penicillium digitatum*, and *Geotrichum citri-aurantii* [78]. Previous studies by Saravanan et al. [79] confirmed the dual mechanism of action of *Bacillus* by performing a dual-culture method to analyze the inhibitory effect of volatile organic compounds/non-volatile organic compounds against *Fusarium oxysporum* f. sp. *cubense*. *Paenibacillus polymyxa*, formerly *Bacillus polymyxa*, was identified as a promising biocontrol agent and alternative to synthetic pesticides in the treatment of the postharvest green mold decay of citrus fruit [80].

The suppressive effect of cultivation broth samples is primarily attributed to the competitive advantage of the producing microorganism’s cells, while in supernatant, the bioactive components responsible for antifungal activity are extracellular metabolites. The changes in the level of antagonistic activity of cultivation broth samples during the cultivation can be related to the multiplication intensity of the producing isolate, shown in Figure 4, where an evident increase in inhibition zones is recorded during the exponential growth phase. On the other hand, during the stationary growth phase when the secondary metabolism activity of the strain is expressed, there is a slight increase in the inhibition zones obtained by the supernatant samples. The highest antagonistic activity is obtained in the last experimental set, which is also expected according to the previously described results for biomass growth. This fact indicates the importance of the maximization of viable cell numbers at the end of cultivation and in the consequent downstream processing steps, as well as during bioproduct storage, distribution, and application in order to provide the maximal possible biomass content as the active component of the biological product at the application site.

The PGP traits of the *Bacillus* sp. BioSol021 cultivation broths, obtained under different agitation and aeration cultivation conditions, as possible bioagents, were examined regarding maize seed germination activity, considering that, from the economic point of view, maize is one of the most important crops hosting mycotoxigenic *A. flavus* strains, representing the entering point of the mycotoxins to the food chain. There are estimations that 11% of the global maize yield is lost every year due to fungal diseases [81]. Furthermore, maize is well known as a crop with shallow roots, making it more sensitive to climate conditions, especially in windy geographic regions, which could contribute to uprooting, resulting in decreased yield [82]. Hence, the development of a strong maize root system during germination and seedling growth is of utmost importance for securing its resistance to harsh physical stresses arising from environmental conditions. Seed germination and seedling growth are strongly affected by the microbes present in the soil where these processes take place and, in the case of maize plants, the microbial presence in the rhizosphere is dominated by *Bacillus* strains [83]. The beneficial traits of *Bacillus* strains regarding seed germination and seedling development include the increase in nutrients availability through enzymatic activity, such as phosphorus, zinc, and potassium solubilization; as well as nitrogen fixation; the production of plant hormones (auxins, including IAA, and cytokinin), siderophores production; as well as the synthesis of antibacterial and antifungal compounds, together with improvements to overall seedling fitness by increasing tolerance to biotic and abiotic stresses [84,85,86,87]. Some of the aforementioned PGP-beneficial traits are proven for the producing microorganism *Bacillus* sp. BioSol021 in this study. When it comes to the effects on seed germination, an increase in seed germination percentage could be observed when comparing *Bacillus* sp. BioSol021 cultivation broths from all four experiments to the negative control, where the maximal seed germination activity (100%) was observed in the B4 experiment, which had the highest values of agitation and aeration rate, and was also characterized by the highest biomass content and antimicrobial activity against the *A. flavus* strains (Table 5). The distribution plot given in Figure 5, as well as data presented in Table 5, give more information about root and shoot length values, indicating a multiple-fold increase in the root and shoot lengths of the maize seeds treated with B4 cultivation broth in comparison with the negative control, as well as with other cultivation broths. For example, 75% of the seeds have shown root lengths larger than 52 mm, 50% of the seeds had root lengths larger than 71 mm, 75% of the germinated seeds had roots longer than 80 mm in the case of the B4 cultivation broth treatment, while similar values for the negative control in terms of root length distribution were 12 mm, 21 mm, and 24 mm, respectively. The highest value of root length was achieved in case of the B4 cultivation broth treatment (96 mm) and was similar in the case of shoot length (27 mm). These results are also confirmed by the mean values of root and shoot length presented in Figure 6, where statistically significant differences in root length could be observed between the B4 cultivation broth treatment and other treatments (including *Bacillus* sp. BioSol021 cultivation broths and the negative control), while in case of shoot length, the B3 and B4 cultivation broth treatments were at the same level of statistical significance according to the Duncan’s multiple range test, simultaneously being significantly higher than the shoot length values in the remaining treatments and negative control at a significance level of 95%. Once again, improvements in seed germination rate and root and shoot development in maize seeds have confirmed the suitability of the applied aeration and agitation conditions in B4 *Bacillus* sp. BioSol021 cultivation, which favors biomass growth, antimicrobial activity against aflatoxigenic *A. flavus*, and PGP traits in maize seeds. Similar results were observed by Yaghoubian et al. [88] when treating maize seed with a cell-free supernatant of *Bacillus* cultivation broth, resulting in an improved germination percentage and rate at different levels of salinity stress, thus contributing to salinity stress alleviation. The possible mechanisms included in the *Bacillus*-related improvement in maize germination and seedling development include the improved activity of hydrolytic enzymes involved in seed germination [89,90], a shift in phytohormone ratios—increased amount of plant growth phytohormones compared with plant growth inhibitors [88]—and the prevention of ROS (reactive oxygen species) accumulation under stress conditions, thus improving antioxidant enzymes activity and preventing damage to seed-stored proteins [91].

## 5. Conclusions

The presented study confirmed the potential of the previously selected *Bacillus* sp. BioSol021 to be used as a biocontrol agent effective against aflatoxigenic *A. flavus* isolates, as well as a possible PGP agent. In the conducted research, biocontrol agent production was scaled up to a 16 L laboratory-bioreactor level to optimize key bioprocess parameters and define a conceptual solution applicable on higher production scales. The results indicated the significant influence of the agitation rate and aeration intensity on biomass production, nutrients, and oxygen consumption, as well as on the antagonistic activity of the producing microorganism against the tested aflatoxigenic fungi. The suppressive effect was explained by the dual mechanism of action, implying the competitive activity of the producing microorganism cells and, on the other hand, antimicrobial activity of the secondary metabolites. The proven ability of the chosen biocatalyst to produce several hydrolytic enzymes and indole compounds indicated the great potential of the created product to be efficient not only as a biocontrol agent but also in expressing many other plant-beneficial activities, which are characteristic for the abovementioned compounds. The beneficial PGP action was confirmed in maize seed germination experiments, where the highest germination percentage together with the highest values of root and shoot length were achieved when treating maize seed using *Bacillus* sp. BioSol021 cultivation broth produced under the cultivation conditions defined at the maximal agitation and aeration rates from the examined range. Further research will be focused on the potential solutions in terms of formulation techniques to achieve the maximal efficiency and desired characteristics of the final biocontrol/PGP product. After the confirmed in vitro activity and laboratory testing, the following investigation steps will also include an estimation and validation of the product efficiency in real conditions by performing field trials.

## Figures and Tables

**Table 1:** Medium composition for the protease activity screening.

Medium Component	Concentration (g/L)
Skim milk	28
Tryptone	5
Yeast extract	2.5
Glucose	1
Agar	15

**Table 2:** Medium composition for the cellulase and xylanase activity screening.

Medium Component	Concentration (g/L)
CMC/xylan	5
Glucose	1
Yeast extract	0.5
KCl	1
K_2_HPO_4_	1
NaNO_3_	1
MgSO_4_·7H_2_O	0.5
Agar	17
pH value	7.0 ± 0.2

**Table 3:** Medium composition for the pectinase activity screening.

Medium Component	Concentration (g/L)
Pectin	10
Yeast extract	0.5
KCl	1
K_2_HPO_4_	1
NaNO_3_	1
MgSO_4_·7H_2_O	0.5
Agar	20
pH value	7.0 ± 0.2

**Table 4:** Growth parameters of *Bacillus* sp. BioSol021 under different cultivation conditions estimated using Gompertz, logistic, and exponential plateau kinetic models.

Parameter	B1	B2	B3	B4
***N_0_*** (log CFU/mL)				
Gompertz	7.240	7.265	7.291	7.237
Logistic	7.241	7.268	7.293	7.240
Exponential plateau	7.239	7.263	7.288	7.234
***N_max_*** (log CFU/mL)				
Gompertz	7.792	8.143	8.208	8.351
Logistic	7.792	8.139	8.204	8.347
Exponential plateau	7.793	8.147	8.212	8.355
***µ*** (1/h)				
Gompertz	0.0541	0.0372	0.0382	0.0482
Logistic	0.0554	0.0387	0.0398	0.0505
Exponential plateau	0.0528	0.0357	0.0367	0.0459
R^2^				
Gompertz	0.9838	0.9719	0.9861	0.9902
Logistic	0.9835	0.9710	0.9854	0.9908
Exponential plateau	0.9842	0.9728	0.9867	0.9895

B1: 0.5 vvm, 100 rpm; B2: 0.5 vvm, 300 rpm; B3: 1.5 vvm, 100 rpm; B4: 1.5 vvm, 300 rpm.

**Table 5:** Germination rate and values of root and shoot length for maize seeds treated with *Bacillus* sp. BioSol021 cultivation broth samples produced under different cultivation conditions.

Parameter	B1	B2	B3	B4	DW
Germination rate (%)	96.00	96.00	96.00	100.00	88.00
Root length					
Mean value (mm)	21.32	21.60	36.64	68.24	17.96
Standard deviation (mm)	11.07	7.62	15.91	16.06	10.49
Maximal value (mm)	38.00	36.00	62.00	95.00	39.00
>75% values larger than… (mm)	14.00	17.00	24.00	52.00	12.00
>50% values larger than… (mm)	22.00	21.00	39.00	71.00	21.00
>25% values larger than… (mm)	31.00	27.00	47.00	80.00	24.00
Shoot length					
Mean value (mm)	5.60	5.72	9.32	11.16	3.60
Standard deviation (mm)	2.47	5.54	8.35	7.89	4.78
Maximal value (mm)	9.00	16.00	24.00	27.00	19.00
>25% values larger than… (mm)	4.00	0.00	2.00	3.00	0.00
>50% values larger than… (mm)	5.00	4.00	7.00	12.00	2.00
>75% values larger than… (mm)	7.00	10.00	15.00	17.00	6.00

B1: 0.5 vvm, 100 rpm; B2: 0.5 vvm, 300 rpm; B3: 1.5 vvm, 100 rpm; B4: 1.5 vvm, 300 rpm; DW–distilled water.

## Data Availability

No new data were created or analyzed in this study. Data sharing is not applicable to this article.
